# Valproic Acid Enhances the Anticancer Effect of L-Ascorbic Acid by Upregulating Sodium-Dependent Vitamin C Transporter 2 in Colorectal Cancer

**DOI:** 10.3390/antiox14070864

**Published:** 2025-07-15

**Authors:** Kawalin Kantawong, Hakim Meutia Diva, Phuong T. Ho, Ahlim Lee, Misae Kiba, Mi-Gi Lee, Hee Kang, Taek-Kyun Lee, Sukchan Lee

**Affiliations:** 1 Department of Integrative Biotechnology, Sungkyunkwan University, Suwon 16419, Republic of Korea; kawalin22@g.skku.edu (K.K.); meutia21@g.skku.edu (H.M.D.); phbeo96@skku.edu (P.T.H.); megimegi7970@skku.edu (A.L.); mmisae@g.skku.edu (M.K.); 2 Gyeonggi Bio Center, Suwon 16229, Republic of Korea; migi@gbsa.or.kr; 3 Humanitas College, Kyung Hee University, 1732 Deogyeong-daero, Yongin 17104, Republic of Korea; shehee@khu.ac.kr; 4 Ecological Risk Research Department, Korea Institute of Ocean Science & Technology, Geoje 53201, Republic of Korea

**Keywords:** SVCT2, vitamin C, pro-oxidant, valproic acid, colorectal cancer

## Abstract

Vitamin C, also known as L-ascorbic acid (AA), functions as a pro-oxidant in cancer at high doses and exerts anticancer effects by generating reactive oxygen species (ROS) and selectively inducing damage to cancer cells. However, AA at low doses promotes cancer cell proliferation. The efficacy of high-dose AA therapy is frequently restricted by inadequate intracellular AA uptake, resulting from low expression of sodium-dependent vitamin C transporter 2 (SVCT2). In this study, we investigated whether valproic acid (VPA), a histone deacetylase inhibitor, could circumvent this constraint by increasing the expression of SVCT2 in colorectal cancer cells, including HCT-116 and DLD-1 with low SVCT2 levels. We found that VPA increased SVCT2 expression in both cell lines. Co-treatment with AA and VPA increased the number of apoptotic cells and enhanced intracellular AA uptake via VPA-upregulated SVCT2, followed by increased ROS production in both cell lines. Furthermore, the combination increased the synergistic anticancer effects and suppressed the hormetic dose response of AA in both cell lines. In a xenograft mouse model, co-treatment decreased tumor size and increased the tumor growth inhibition ratio compared to treatment with AA or VPA alone. Accordingly, VPA treatment enhanced SVCT2 expression in colorectal cancer cells, suppressed the hormetic dose-response effect of AA, and improved the potential of high-dose AA therapy as an anticancer agent.

## 1. Introduction

Vitamin C (L-ascorbic acid, AA), which is well known for its antioxidant properties, functions as a pro-oxidant in cancer cells, inducing cytotoxicity with high intracellular concentration [1,2,3]. The anticancer properties of AA reportedly involve inhibition of cell growth and proliferation via the generation of reactive oxygen species (ROS) and hydrogen peroxide-mediated mechanisms [4,5,6,7]. ROS induces oxidative stress and cellular damage depending on the metabolic circumstances and redox status of cancer cells [8,9]. The half-maximal effective concentration (EC50) of AA varies between cancer and healthy cells; normal cells have an EC50 of more than 20 mM for vitamin C, while cancer cells have an EC50 of 1–10 mM, resulting in selective cytotoxicity [1,10]. The anticancer effect of high-dose AA has been extensively explored in several cancer types in vitro, in vivo, and in clinical trials [2,3,11,12,13,14].

Linus Pauling and Ewan Cameron first recognized the potential of AA as a cancer therapeutic agent in 1976 and demonstrated that high-dose AA therapy prolonged the survival of patients with cancer [15,16]. Nevertheless, a subsequent study conducted at the Mayo Clinic reported that AA did not induce any notable beneficial effects in patients with cancer [17,18]. This discrepancy could be explained by the sodium-dependent vitamin C transporter family 2 (SVCT2), which regulates AA intake and may play an important role in determining the efficacy of high-dose AA therapy [19,20,21]. A study exploring the hormetic proliferation responses of colorectal cancer cell lines to AA found that high doses of AA exerted anticancer effects. However, in low-SVCT2-expressing cells, low-dose AA (<10 mM) enhanced cancer cell proliferation. Conversely, all AA doses (10 μM to 2 mM) induced cancer cell death in cell lines with high SVCT2 expression [22]. These findings highlight the need to improve AA transport to enhance its therapeutic efficacy. Increasing SVCT2 expression may be crucial for enhancing the efficacy of high-dose AA therapy, considering the heterogeneity in SVCT2 expression among patients.

Valproic acid (VPA) is a potential modulator of SVCT2 and induces AA uptake by regulating HDAC2 expression in a neuronal cell line and the mouse brain [23]. VPA is a short-chain fatty acid and class I histone deacetylase (HDAC) inhibitor that has traditionally been used to treat epilepsy and other neurological disorders [24,25,26]. The anticancer properties of VPA have also been examined in a wide range of cancer types, including ovarian, breast, pancreatic, lung, prostate, oral, and colorectal cancers, in vitro, in vivo, and in clinical trials [10,27,28,29,30,31,32,33]. VPA inhibits tumor growth by modifying the expression of genes associated with the HDAC family [28,34]. VPA reportedly suppresses the expression of class I HDAC, resulting in the upregulation of p21, a tumor suppressor, and acetylation and activation of p53 and p65, which stimulates apoptosis in liver cancer cells and prevents cancer cell proliferation, migration, and invasion [35]. VPA-induced demethylation of DNA and hyperacetylation of histones to their relaxed form, which is associated with increased gene transcription linked to cancer growth in breast cancer [36].

Increasing SVCT2 expression in low-SVCT2-expressing cancer cells is crucial for improving the efficacy of high-dose AA therapy, given that SVCT2 levels are highly associated with anticancer activity. In this study, we examined the anticancer effects of VPA and AA. We hypothesized that VPA enhanced the anticancer effects of AA by increasing the expression of SVCT2 in colorectal cancer cells. Our results showed that VPA enhanced the anticancer effects of AA by upregulating SVCT2 expression, resulting in increased AA uptake, ROS generation, and apoptotic cells in low-SVCT2-expressing colorectal cancer, HCT-116 and DLD-1, compared with AA treatment alone (without VPA). In a xenograft mouse model, co-treatment with AA and VPA significantly reduced tumor growth. These results suggest that VPA may function as an SVCT2 inducer, providing a promising avenue for future investigations into its potential role in enhancing high-dose AA therapy in patients with cancer exhibiting low SVCT2 expression.

## 2. Materials and Methods

### 2.1. Cell Culture and Reagents

Human colorectal cell lines with low SVCT2 expression, HCT-116 and DLD-1, were purchased from the Korean Cell Line Bank (KCLB). The cells were cultured in RPMI-1640 medium supplemented with L-glutamine (CM059-050; GenDEPOT, Katy, TX, USA), 10% fetal bovine serum (FBS) (Gibco, Waltham, MA, USA), and 1% penicillin-streptomycin solution (SV30010; Hyclone, Logan, UT, USA). MC38, a mouse colorectal cancer cell line, was procured from the National Cancer Center (Goyang, Republic of Korea) and cultured in DMEM containing high glucose, 4.0 mM L-glutamine, sodium pyruvate (CM002-050; GenDEPOT, Katy, TX, USA), 10% FBS, and 1% penicillin-streptomycin solution at 37 °C in a humidified incubator with 5% CO_2_. Vitamin C and AA were purchased from Huons Biopharma (Seongnam, Republic of Korea), and VPA was purchased from Sigma-Aldrich (St. Louis, MO, USA).

### 2.2. Cell Viability Assay

Cell viability was measured using the D-Plus™ CCK cell viability assay kit (Dongin Biotech, Seoul, Republic of Korea). In brief, cells (1 × 10^4^/well) were seeded and cultured in 96-well plates for 24 h. Subsequently, cells were treated with AA and VPA in RPMI-1640 medium with L-glutamine with 5% FBS and 1% penicillin-streptomycin solution for 48 h at 37 °C and under 5% CO_2_. The cells were then washed with Dulbecco’s phosphate-buffered saline (DPBS; LB001-02; Gyeongsan, Republic of Korea). To measure cell viability, 10 μL of the CCK-8 solution in 90 μL DPBS was added to each well; cells were incubated at 37 °C under 5% CO_2_ for 1 h. The absorbance was measured at 450 nm using a microplate reader (Epoch, San Jose, CA, USA).

### 2.3. Crystal Violet Assay

To examine anticancer effects, a crystal violet assay was performed using stained cells attached to cell culture plates. Briefly, cells (6 × 10^5^/well) were seeded, cultured in 6-well plates, and incubated for 24 h. Next, the cells were treated with AA and VPA in RPMI-1640 medium with L-glutamine, 5% FBS, and 1% penicillin-streptomycin solution for 48 h at 37 °C under 5% CO_2_. The cells were stained with a 0.5% crystal violet staining solution, and the plate was placed on a shaker at room temperature and incubated for 30 min. Subsequently, the staining solution was removed, the cells were washed with distilled water, the plate was reversed on the bench, air-dried at room temperature, and the stained cells were observed. The crystal violet staining was directly proportional to the cell biomass attached to the plates.

### 2.4. Combination Index (CI) Analysis

To determine the effect of using combinations of AA and VPA in HCT-116 and DLD-1 cells, the combination index (CI) was calculated using CompuSyn software (Version 1.0, CompuSyn, Inc., and Paramus, NJ, USA). In a matrix design, cells were exposed to various concentrations of VPA (0.25, 0.5, 1, and 2 mM) combined with AA (0.1, 0.5, 1, and 2 mM). Cell viability was measured using the CCK-8 assay and converted to fractional inhibition (Fa) using the formula: Fa = 1 − (viability of treated cells/viability of untreated control cells). For the calculation of CI values, the Fa values and drug concentrations for single and combination treatments were entered into the CompuSyn software. CI < 1 indicates synergy for a compound pair, CI < 0.3 indicates strong synergy, and CI > 1 indicates antagonism. The Fa-CI plots were generated using GraphPad Prism 8.0 (GraphPad Software, San Diego, CA, USA).

### 2.5. Quantitative Real-Time PCR (qPCR) Analysis

Total RNA was extracted from the cells using the TRI reagent (TR118; Molecular Research Center, Cincinnati, OH, USA) according to the manufacturer’s guidelines. RNA concentration was determined by measuring the absorbance at 260 nm. cDNA was synthesized using the CellScript cDNA Master Mix (CDS-200; Cellsafe, Yongin, Republic of Korea) according to the manufacturer’s protocol. qPCR was performed using the TB Green Premix Ex Taq (TaKaRa Bio, Otsu, Japan). Data were analyzed using Rotor-Gene O series software (version 2.3.1; Qiagen, Hilden, Germany). The following genes were amplified using the indicated primer sequences, *hSVCT2*: forward 5′-TCTTTGTG CTTGGATTTTCGAT-3′ and reverse 5′-ACGTTCAACACTTGATCGATTC-3′; *mSVCT2*: forward 5′-AACGGCAGAG CTGTTGGA-3′ and reverse 5′-GAAAATCGTCAGCATGGCAA-3′; *hβ-actin*: forward 5′-CATCC TGCGTCTGGACCT-3′ and reverse 5′-TAATGTCACGCA CGATTTCC-3′; and *mβ-actin*: forward 5′-ATCCTCTTCCTCCCTGGA-3′ and reverse 5′-TTCATGGATGC CACAGGA-3′. The relative gene expression was then determined using the formula 2^−ΔΔCt^.

### 2.6. Western Blotting

Proteins were extracted from the cells and tissues of mice using a Pro-Prep Protein Extraction Kit containing a protease inhibitor cocktail (17081; iNtRON, Sungnam, Republic of Korea). Protein concentrations were measured using a Bradford Protein Assay Plus Kit (P7200-050; GenDepot, Katy, TX, USA). In total, 25 mg of protein was heat-denatured in sample buffer for 10 min. Protein samples were loaded onto 10% sodium dodecyl sulfate-polyacrylamide gels and transferred onto polyvinylidene difluoride membranes. The membranes were blocked with 5% skimmed milk in Tris-buffered saline with Tween 20 (TBST) at room temperature for 2 h. Subsequently, the membranes were incubated overnight at 4 °C with anti-SVCT2 (1:500) (A6740; ABclonal, Wuhan, China), anti-p21 (1:2500) (bs-10129R; Bioss, Beijing, China), anti-caspase-3 (1:2500) (#9662S; Cell Signaling Technology, Danvers, MA, USA), and anti-beta-actin (1:2000) (NB600-501; Novus biologics, Centennial, CO, USA) antibodies. After four washes with TBST, the membranes were incubated with secondary anti-rabbit antibodies for 2 h at room temperature. After additional washing with TBST, immune-reactive bands were detected using West-Q Pico Dura ECL Solution (W3653; GenDepot, Katy, TX, USA) and visualized using the iBright™ CL1500 Imaging System (Invitrogen, Waltham, MA, USA).

### 2.7. AA Uptake

The cells were harvested and incubated with VPA for 48 h and AA for 4 h. A commercially available ascorbic acid kit (ab65346; Abcam, Waltham, MA, USA) was used to determine the intracellular AA concentration. In this assay, a proprietary catalyst oxidizes AA to produce products that interact with the AA probe, thereby generating a color. The color developed within 3 min and was stable for 1 h. Absorbance was measured at 570 nm (Epoch, San Jose, CA, USA). The reaction mixture was prepared according to the manufacturer’s instructions. AA concentrations in the cells were analyzed compared to AA standard curve as described in the kit, and the equation ‘concentration = As/Sv’ (where As is the AA amount determined from the standard curve and Sv is the sample volume added to the sample wells) was used as suggested in the kit description.

### 2.8. Detection of ROS Generation

ROS formation was assessed by fluorescence microscopy using a DCFDA/H2DCFDA-Cellular ROS Assay kit (Abcam, Cambridge, UK) according to the manufacturer’s protocol. Briefly, the cells were seeded in 24-well plates at a density of 6 × 10^4^ cells per well in RPMI-1640 medium with L-glutamine, 5% FBS, and 1% penicillin-streptomycin solution, followed by overnight incubation at 37 °C and under 5% CO_2_. Subsequently, the cells were treated with 1 mM VPA for 48 h. Then, cells were stained with 20 μM dichloro-dihydro-fluorescein diacetate (DCFH-DA) for 30 min at 37 °C. Cells were treated with AA (0.5 mM) for 4 h. After treatment, the cells were trypsinized using trypsin-EDTA (1×) and 0.25% (CA014-010; GenDepot, Katy, TX, USA) and washed twice with DPBS. ROS production was detected by flow cytometry (Navios EX Flow Cytometer, Beckman Coulter Life Sciences, Indianapolis, IN, USA). DCF was excited using a 488 nm laser and detected at 535 nm (FL1). Flow cytometry data were analyzed using Kaluza Analysis Software version 2.4 (Beckman Coulter, Indianapolis, IN, USA).

### 2.9. Apoptosis Assay

For apoptosis detection, flow cytometry was performed using BD Pharmingen™ FITC Annexin V Apoptosis Detection Kit I (BD Biosciences, Franklin Lakes, NJ, USA) according to the manufacturer’s protocol. Briefly, cells were seeded at a density of 6 × 10^5^ cells/well in 6-well plates. Then, cells were treated with AA and VPA in RPMI-1640 media with L-glutamine, 5% FBS, and 1% penicillin-streptomycin solution for 48 h at 37 °C and under 5% CO_2_. The cells were trypsinized with trypsin-EDTA, washed twice with DPBS, resuspended in 1× binding buffer, and stained with annexin V and propidium iodide. Following incubation for 15 min at room temperature in the dark, the samples were immediately analyzed by flow cytometry (Navios EX Flow Cytometer, Beckman Coulter Life Sciences, Indianapolis, IN, USA).

### 2.10. Knockdown of SVCT2 Activity Using Lentivirus

shRNA oligonucleotide design, lentiviral particle production, and target cell infection were performed as described by Addgene “https://www.addgene.org/protocols/plko/ (accessed on 20 January 2025)”. The target gene knockdown sequences for shSVCT2 and scrambled controls were obtained from Sigma-Aldrich.

HCT-116 cells were seeded in RPMI-1640 medium containing L-glutamine, 5% FBS, and 1% penicillin-streptomycin solution at 37 °C under 5% CO_2_. Upon reaching 50% confluency, cells were infected with lentiviral particles in the presence of 8 μg/mL polybrene (sc-134220; Santa Cruz Biotechnology, Dallas, TX, USA). Forty-eight hours post-transfection, cells were selected using a growth medium containing 2 μg/mL puromycin (ant-pr-1; InvivoGen, San Diego, CA, USA) to establish stably transfected cell lines. Western blotting was performed to determine the efficiency of shSVCT2 knockdown.

### 2.11. Animal Experiment

Six-week-old male C57BL6 mice were purchased from Dae Han Bio (Link, Chungbuk, Republic of Korea). Mice were housed in a pathogen-free environment at 24 °C under a 12 h/12 h light-dark cycle. This study was approved by the Administrative Panel of the Laboratory Animal Research Center at Sungkyunkwan University School of Medicine (approval number: SKKUIACUC2023-04-26-1). To determine the anticancer effects in vivo, 1 × 10^6^ MC38 cells were subcutaneously injected into the dorsal surface of mice to establish a subcutaneous xenograft tumor mouse model. Tumor volume was calculated with the formula: tumor volume = (length) × (width)^2^ × 0.5. In the model mice, when the tumor volumes ranged between 20 and 30 mm^3^, the mice were randomly allocated into four groups (*n* = 3): control, AA, VPA, and co-treatment with AA and VPA. This randomization ensured comparable baseline tumor characteristics across all groups prior to treatment initiation. AA (4 g/kg) was administered via intraperitoneal injection (IP) every 2 days to achieve high plasma concentrations [11], and 200 mg/kg VPA, which elevates SVCT2 expression in mouse brains [23], was administered intraperitoneally daily for 14 days. The relative tumor volume (RTV) was calculated as follows: RTV = tumor volume on the measured day/tumor volume on day 0. Tumor volumes were measured, and the percentage of tumor growth inhibition (%TGI) was calculated as follows: %TGI = [1 − (RTV treatment group/RTV control group)] × 100.

### 2.12. Statistical Analysis

Data were analyzed using GraphPad Prism 8.0 (GraphPad Software, San Diego, CA, USA). Results are presented as the mean ± standard error of the mean (SEM). For comparisons among three or more groups, one-way or two-way ANOVA followed by Tukey’s Honest Significant Difference (HSD) post hoc test was applied for multiple comparisons, as appropriate. Differences were considered statistically significant at * *p* < 0.05, ** *p* < 0.01, and *** *p* < 0.001; ns, not significant.

## 3. Results

### 3.1. Co-Treatment with AA and VPA Exerts Synergistic Anticancer Effects

**Figure 1:**
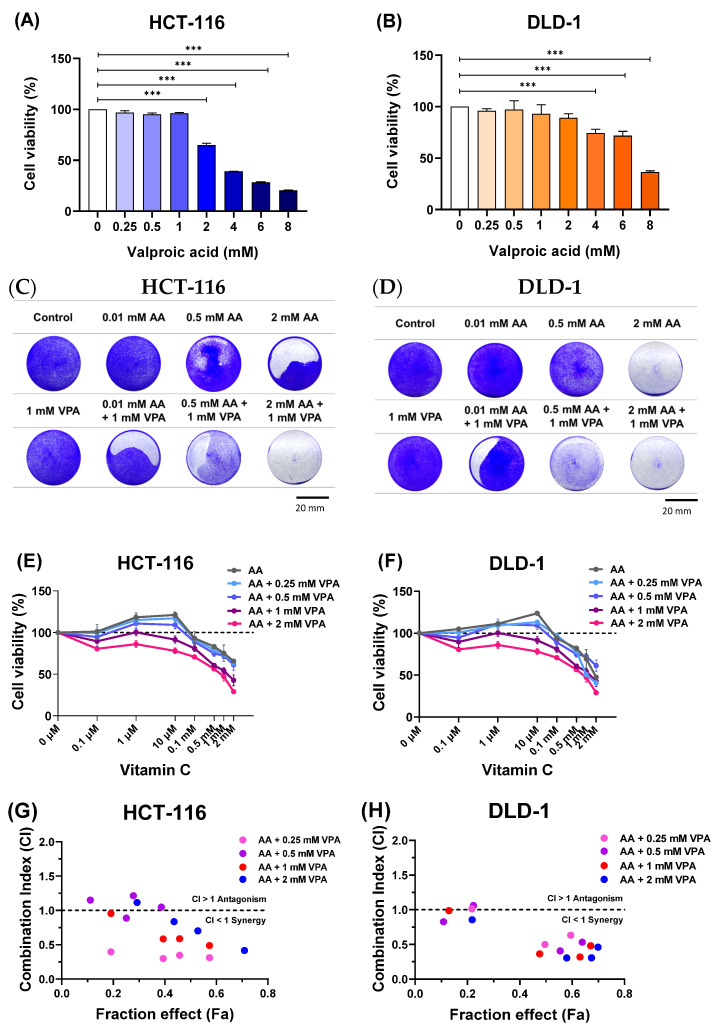
Co-treatment with AA and VPA increased the anticancer effects. The cytotoxic effects of VPA were assessed on HCT-116 and DLD-1 cells (**A**,**B**). Co-treatment with AA and VPA for 48 h enhanced the anticancer effects of AA. Crystal violet staining assays were conducted following the co-treatment in both cell lines (scale bar = 20 mm) (**C**,**D**). VPA could suppress the hormetic dose response of AA in the low-SVCT2-expressing cell lines HCT-116 and DLD-1 (**E**,**F**). The fraction affected (Fa) versus combination index (CI) plot after co-treated with AA and VPA in both cell lines (**G**,**H**). A one-way ANOVA was carried out to determine significant differences, followed by Tukey’s HSD post hoc test: *** *p* < 0.001.

To determine the cytotoxic effects of VPA on HCT-116 and DLD-1 cells, the cells were treated with VPA (0.25–8 mM) for 48 h. Subsequently, cell viability was evaluated using CCK-8 assays. Treatment with 0.25–1 mM VPA did not induce cytotoxicity, but VPA higher than 1 mM was toxic to HCT-116 cells (Figure 1A). Similarly, treatment with 0.25–2 mM VPA did not induce cytotoxicity in DLD-1 cells, whereas VPA concentrations greater than 2 mM reduced the cell viability (Figure 1B). Next, the cells were co-treated with AA (0.1 μM–2 mM) and VPA (1 mM) for 48 h, followed by crystal violet staining. In both cell lines, co-treatment with AA and VPA increased the anticancer effects, especially when 1 mM VPA was combined with low doses of AA (0.01 and 0.5 mM) in both cell lines (Figure 1C,D). Additionally, co-treatment with AA and VPA suppressed the hormetic dose-dependent effect of AA. Treatment with gradient concentrations of AA (0.1 μM–2 mM) alone showed that high doses of AA (>1 mM) reduced cell viability, whereas low doses of AA (<0.5 mM) promoted cancer cell growth in both cell lines. Conversely, co-treatment with low-dose AA and VPA reduced cell viability compared with treatment with AA alone. Additionally, co-treatment with high doses of AA and VPA decreased the viability of both cell lines (Figure 1E,F). The combination index (CI) was calculated using CompuSyn software to determine the synergistic of the combined treatment with AA (0.1, 0.5, 1, and 2 mM) and VPA (0.25, 0.5, 1, and 2 mM) in both cell lines. The fraction effect and CI for the combined exhibited a synergic anticancer effect (CI < 1) (Figure 1G,H). Collectively, these findings revealed that VPA suppressed the hormetic dose-response of AA, which has a synergistic anticancer effect in the low-SVCT2-expressing cell lines, HCT-116 and DLD-1.

### 3.2. VPA Upregulates SVCT2 Expression and Induces AA Uptake in Colorectal Cancer Cell Lines

**Figure 2:**
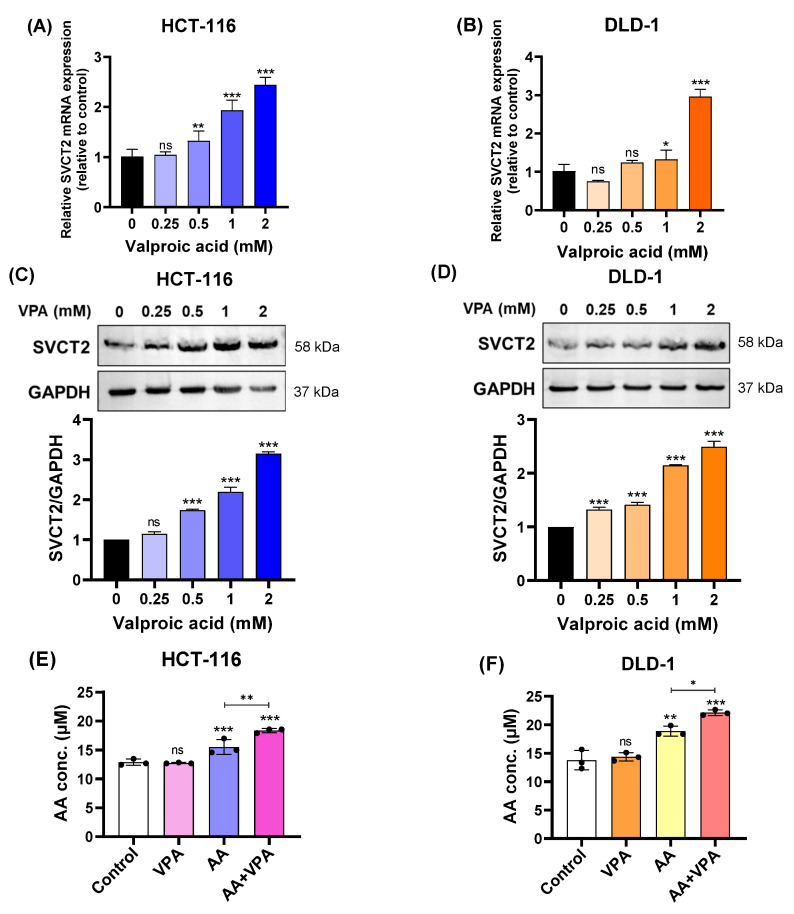
VPA upregulated SVCT2 expression and induced *AA* uptake in colorectal cancer cell lines. The cells were treated with VPA for 48 h in HCT-116 and DLD-1 cells. *SVCT2* mRNA expression was determined using qPCR (**A**,**B**). SVCT2 protein expression was analyzed by western blotting (**C**,**D**). Both cell lines were treated with VPA for 48 h, followed by treatment with AA for 4 h to evaluate the intracellular AA concentrations (**E**,**F**). One-way ANOVA was performed to determine significant differences, followed by Tukey’s HSD post hoc test: * *p* < 0.05, ** *p* < 0.01, and *** *p* < 0.001; ns: not significant.

In our study, we treated HCT-116 and DLD-1 colorectal cancer cell lines with low SVCT2 expression with VPA (0.25–2 mM) for 48 h and determined the expression levels of the SVCT2 gene and protein. According to the qPCR results, *SVCT2* mRNA expression increased in both HCT-116 and DLD-1 cells in a concentration-dependent manner (Figure 2A,B). Similarly, western blot analysis revealed that VPA treatment enhanced SVCT2 protein levels in both cell lines in a concentration-dependent manner (Figure 2C,D). To elucidate the role of SVCT2 in vitamin C transport, both cell lines were treated with 1 mM VPA for 48 h, followed by treatment with 0.5 mM AA for 4 h. An ascorbic acid assay kit (Abcam) was used to measure intracellular AA concentrations. In both HCT-116 and DLD-1 cells, co-treatment with AA and VPA significantly increased intracellular AA levels compared to AA treatment alone (Figure 2E,F). Accordingly, VPA treatment increased SVCT2 expression, which induced AA uptake in the HCT-116 and DLD-1 colorectal cancer cell lines with low SVCT2 expression.

### 3.3. Co-Treatment with AA and VPA Induces ROS Generation and Increases Apoptotic Response

**Figure 3:**
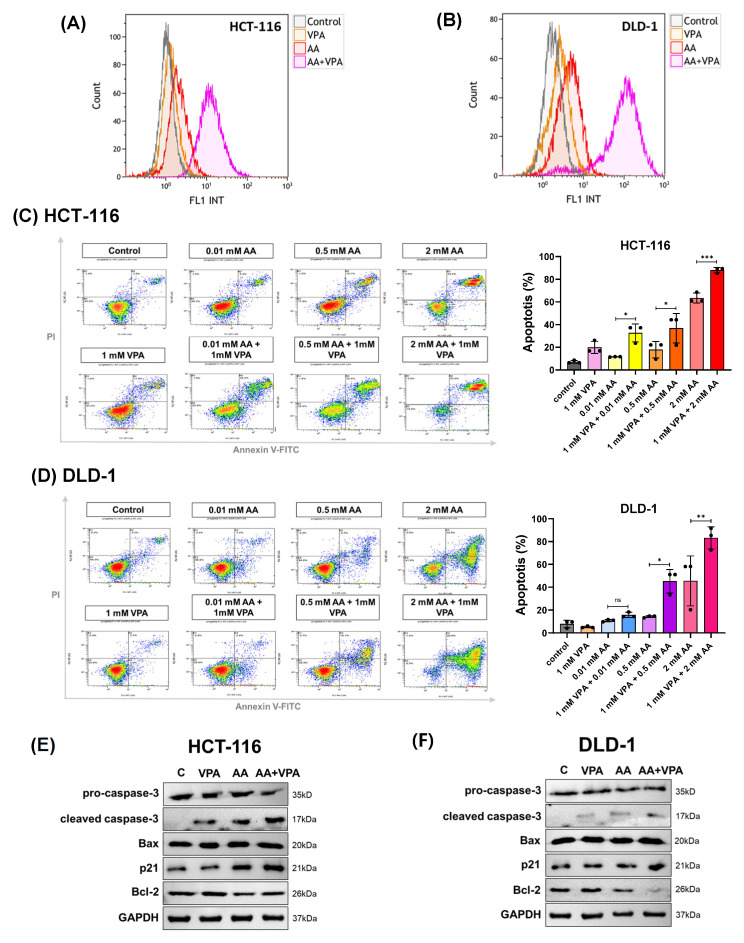
Co-treatment with AA and VPA induced ROS generation and increased the apoptotic response. ROS generation was measured by flow cytometer after the cells were co-treated with AA and VPA in HCT-116 and DLD-1 cells (**A**,**B**). Apoptosis was investigated in both cell lines after co-treatment using a flow cytometer with annexin V and propidium iodide (PI) labeling (**C**,**D**), and the expression of apoptosis-related proteins was determined by western blotting (**E**,**F**). One-way ANOVA was performed to determine significant differences, followed by Tukey’s HSD post hoc test: * *p* < 0.05, ** *p* < 0.01, and *** *p* < 0.001; ns: not significant.

High levels of AA increase ROS generation, which damages cancer cells and promotes cancer growth. In this study, ROS production was measured by flow cytometry after labeling the cells with DCFDA. In both HCT-116 and DLD-1 cells, co-treatment with AA (0.5 mM) and VPA (1 mM) induced greater ROS production than treatment with AA or VPA alone (Figure 3A,B). To measure apoptosis, cells were stained with Annexin V and propidium iodide and subjected to flow cytometry. Compared to treatment with AA or VPA alone, co-treatment with AA and VPA increased the percentage of apoptotic cells in a concentration-dependent manner in both cell lines (Figure 3C,D). Western blotting analysis was performed to determine the expression of apoptosis-related proteins. Compared to treatment with either AA or VPA alone, co-treatment with 0.5 mM AA and 1 mM VPA enhanced the levels of cleaved caspase-3, Bax, and p21 proteins and decreased those of pro-caspase-3 and Bcl-2 in both cell lines (Figure 3E,F). Taken together, these results suggest that VPA enhances the anticancer effects of AA in the low-SVCT2-expressing colorectal cancer cell lines, HCT-116 and DLD-1, by stimulating ROS production and inducing cell apoptosis.

### 3.4. VPA Induces SVCT2 Expression in SVCT2-Knockdown HCT-116 Colorectal Cancer Cells

**Figure 4:**
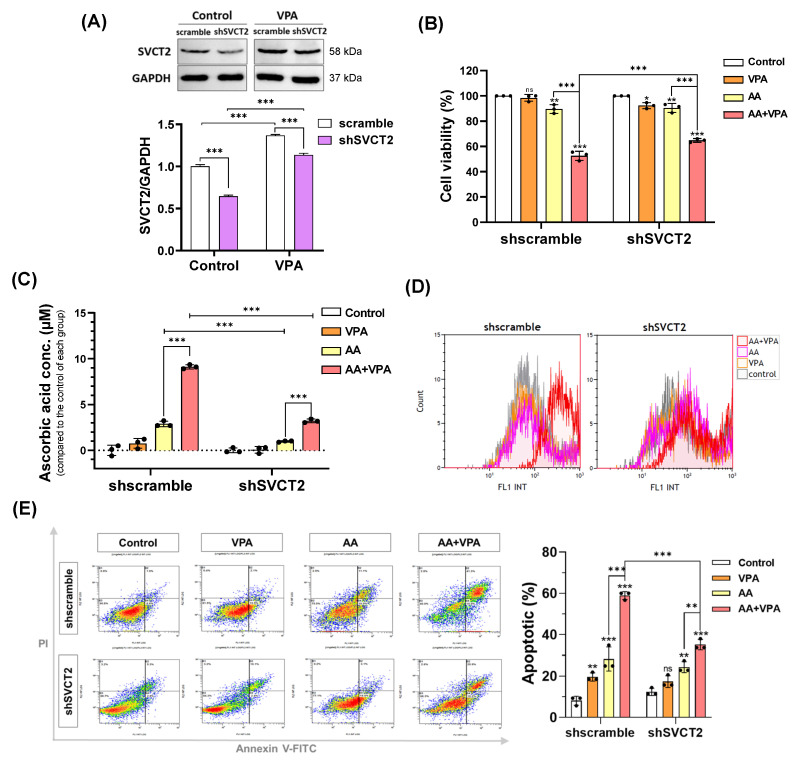
VPA induces SVCT2 expression in SVCT2-knockdown HCT-116 colorectal cancer cells. HCT-116 shSVCT2 cells were treated with VPA for 48 h, and SVCT2 protein expression was examined by western blotting (**A**). The cells were co-treated with AA and VPA to determine their anticancer effects (**B**), intracellular AA concentrations (**C**), ROS generation (**D**), and apoptosis in HCT-116 shSVCT2 cells (**E**). Two-way ANOVA was performed to determine significant differences, followed by Tukey’s HSD post hoc test: * *p* < 0.05, ** *p* < 0.01, and *** *p* < 0.001; ns: not significant.

VPA reportedly exerts anticancer effects against several types of cancer cells, including colorectal cancer cell lines HCT-116 and DLD-1 (Figure 1A,B). VPA decreased the viability of HCT-116 cells at concentrations >1 mM and DLD-1 cells at concentrations >2 mM. In addition, AA exhibited anticancer properties at high doses. In the current study, our findings indicated that co-treatment with AA and VPA increased the synergistic anticancer effects. To determine whether VPA enhances the anticancer effects of AA by upregulating the expression of SVCT2. HCT-116 cells were infected with lentiviral particles containing shSVCT2, with the average knockdown efficiency of approximately 35.6% at the protein level compared to shscramble control. HCT-116 shSVCT2 cells treated with 1 mM VPA for 48 h exhibited a significant upregulation of SVCT2 expression compared to untreated HCT-116 shSVCT2 cells (Figure 4A). Moreover, in HCT-116 shSVCT2 cells, co-treatment with AA (0.5 mM) and VPA (1 mM) significantly decreased the anticancer effect (Figure 4B), reduced intracellular AA uptake (Figure 4C), diminished ROS generation (Figure 4D), and lowered apoptotic rates (Figure 4E) compared to shscramble control cells. These findings suggest that the anticancer activity of AA is dependent on SVCT2 expression levels and that VPA enhances this effect by upregulating SVCT2 expression in the colorectal cancer cells.

### 3.5. VPA Upregulates SVCT2 Expression in the Tumor, and Co-Treatment with AA and VPA Exerts an Antitumor Effect in the Xenograft Mouse Model

**Figure 5:**
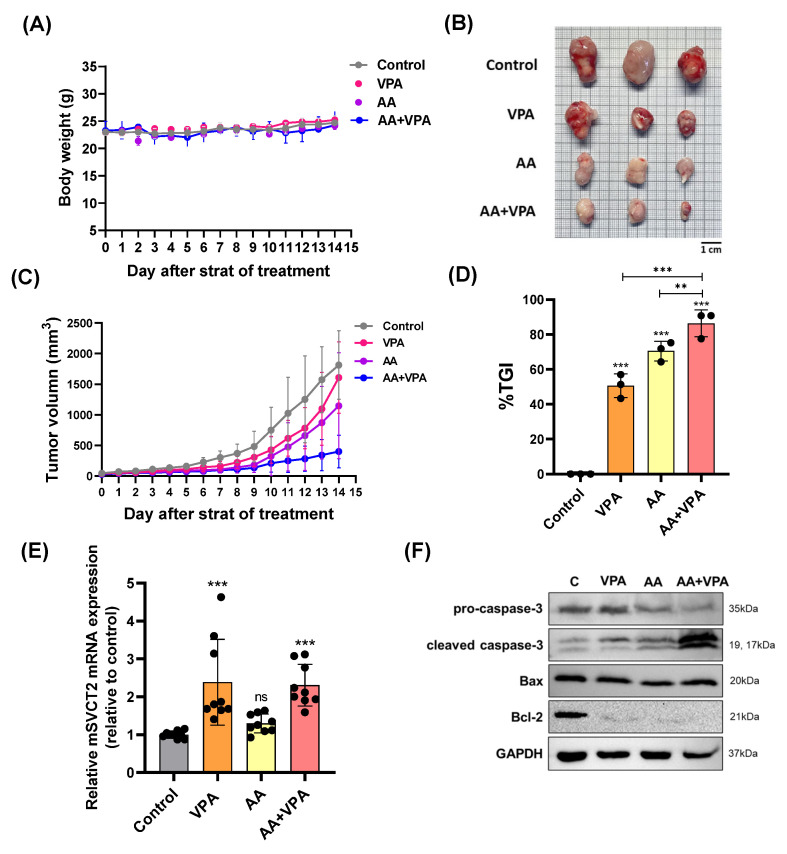
VPA upregulates *mSVCT2* expression in tumor, and co-treatment with AA and VPA exerts an antitumor effect in mouse xenograft model. The antitumor effect of co-administration with AA and VPA was measured in the mouse xenograft model (*n* = 3), AA was administered every 2 days for 14 days and VPA was daily administered for 14 days. The mice were daily measured for body weight (g) (**A**). After administering, we observed tumor size (Scale bar = 1 mm) (**B**), tumor volume (mm^3^) (**C**), and tumor growth inhibition ratio (%TGI) was analyzed for all the treatment groups (**D**). *mSVCT2* gene expression in the tumor was investigated by qPCR (**E**). Apoptotic-related markers were determined by western blotting (**F**). A one-way ANOVA was carried out to determine significant differences, followed by Tukey’s HSD post-hoc test: ** *p* < 0.01, and *** *p* < 0.001, and ns: not significant.

To evaluate the antitumor effects of co-treatment with AA and VPA in a xenograft mouse model, AA (4 g/kg body weight) was administered via intraperitoneal injection every 2 days for 14 days, whereas VPA (200 mg/kg body weight) was administered daily via intraperitoneal injection for 14 days. There were no significant changes in body weight in all the treatment groups (Figure 5A). Furthermore, co-treatment with AA and VPA significantly reduced tumor volume (mm^3^), tumor size, and % TGI compared to treatment with AA or VPA alone (Figure 5B–D). Furthermore, treatment with VPA and the co-treatment increased *mSVCT2* gene expression in the tumor approximately 2.3 times compared to the control group; the higher levels of SVCT2 in the tumor might increase the cancer effects of AA. (Figure 5E). In addition, co-treatment with AA and VPA enhanced the expression of apoptosis-related proteins, including cleaved caspase-3 and Bax. In contrast, the co-treatment reduced the levels of Bcl-2 and pro-caspase-3 within the tumor compared to AA or VPA treatment alone (Figure 5F).

## 4. Discussion

High-dose AA therapy may exert anticancer effects by acting as a pro-oxidant and inducing the generation of reactive ROS generation in cancer cells. The efficacy of this therapy depends on the expression of SVCT2 in cancer cells and patients with cancer [11,22,35,37,38,39]. SVCT2 is a key protein transporter that regulates intracellular AA levels [11,19,20]. In colorectal cancer cell lines with high SVCT2 expression, both low and high doses of AA have been reported to reduce cell viability. High-dose AA (>1 mM) could exert an anticancer effect in low-SVCT2-expressing colorectal cancer cell lines, whereas treatment with low-dose AA (<10 μM) promotes cancer cell proliferation [22]. These findings suggest that SVCT2 expression in cancer cells plays a role in improving the therapeutic effects of high-dose AA anticancer therapy. In this study, we aimed to identify an inducer capable of upregulating SVCT2 expression, thereby promoting high-dose intracellular AA uptake in low-SVCT2-expressing cancer cells to achieve enhanced anticancer effects.

In human breast cancer cell lines, AA treatment combined with magnesium supplementation had a more effective anticancer effect than AA treatment alone, specifically by inhibiting the hormetic response to AA at low doses. Although magnesium supplementation improves the functional activity of SVCT2, it does not upregulate SVCT2 expression [11]. VPA induces AA uptake by decreasing HDAC2 levels, which increases SVCT2 mRNA and protein expression. This regulation correlates with the YY-1 transcription factor, which promotes SLC23A2 promoter activity in a neural model [23]. Additionally, VPA suppresses cell proliferation in various cancer types by affecting the activity of HDAC, thereby inducing apoptosis and cell cycle arrest [36,40,41,42]. Furthermore, VPA alone and in combination with other chemotherapeutic agents has exhibited notable anticancer effects in animal model studies and clinical trials [40,43,44]. VPA increases the expression of genes involved in tumor differentiation and progression that are regulated by the ERK-AP-1 pathway, including growth-associated protein-43 (GAP-43) and Bcl-2 [45]. Additionally, VPA decreased head and neck squamous cell carcinoma cells proliferation by upregulating p21 and inducing G0/G1 cell cycle arrest [46]. In a human ovarian cancer cell line (OVCAR-3), VPA reduced cell proliferation by downregulating genes responsible for G1-to-S phase transition and upregulating those involved in G1 phase arrest [47]. Regarding the anticancer effect in various cancer types and SVCT2 regulation in the neuronal model of VPA, VPA could serve as an SVCT2 inducer, which induces high-dose AA treatment and increases the synergistic anticancer effect of AA.

**Figure 6:**
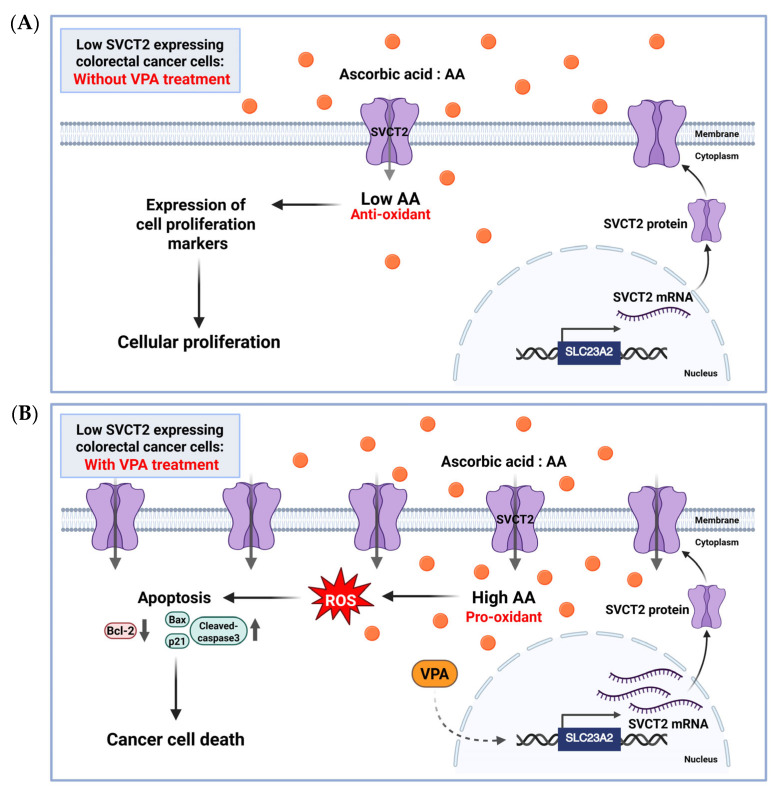
The proposed role of AA and VPA in anticancer effects involves the induction of SVCT2 expression. AA (orange dots) treatment in low SVCT2-expressing colorectal cancer cells without VPA treatment, at low intracellular concentrations, AA functions primarily as an antioxidant, promoting cellular proliferation and supporting cancer growth (**A**). In contrast, in low SVCT2-expressing colorectal cancer cells, treatment with VPA enhances SVCT2 expression, leading to increased intracellular uptake of AA. This higher amount of AA changes its role to a pro-oxidant, causing the production of ROS, leading to cancer cell death through apoptosis (**B**). Created in https://BioRender.com.

To our best knowledge, no studies have explored the stimulation of SVCT2 expression by VPA alone or in combination with AA in colorectal cancer cells. In the current study, we investigated co-treatment with AA and VPA exerted synergistic anticancer effects by upregulating SVCT2 expression in low-SVCT2-expressing colorectal cancer cell lines. Cho et al. 2018 [22] examined SVCT2 expression in various colorectal cancer cell lines and found that HCT-116 and DLD-1 cells exhibited low SVCT2 expression. Therefore, we evaluated the anticancer effects of AA and VPA on two colorectal cancer cell lines with low SVCT2 expression, HCT-116 and DLD-1 cells. Our data revealed that VPA treatment is associated with elevated SVCT2 levels in both cell lines. Co-treatment with AA and VPA substantially enhanced intracellular AA uptake compared with AA treatment alone, which promoted ROS generation and increased apoptotic cells in both cancer cell lines. Furthermore, the co-treatment also enhanced the expression of the apoptotic proteins cleaved caspase-3, Bax, and p21, while suppressing the expression of the anti-apoptotic proteins Bcl-2 and pro-caspase-3. Moreover, our findings indicated that combining AA and VPA had a synergistic anticancer effect on HCT-116 and DLD-1 cells. Notably, co-treatment with low-dose AA and VPA reduced the viability of both cell lines, which suppressed the hormetic proliferation response to AA in both low-SVCT2-expressing cell lines. According to the anticancer efficiency of both AA and VPA, we investigated whether decreased cancer cell viability was associated with increased SVCT2 expression, as determined in shSVCT2 HCT-116 cells, VPA treatment significantly reduced SVCT2 expression levels in the compared to shscramble control cells. In HCT-116 shSVCT2 cells, co-treatment with AA and VPA resulted in decrease intracellular uptake of AA, ROS generation, apoptotic rates, and diminished anticancer effects. Therefore, treatment with VPA in low-SVCT2-expressing cells upregulated SVCT2 expression level, which induced intracellular AA uptake and acts as a pro-oxidant in the cancer cells by generating ROS and enhanced anticancer effect of high dose AA therapy (Figure 6).

A previous study found that VPA enhanced the expression of mSVCT2 protein, mRNA, and heterogeneous nuclear RNA (hnRNA) in mice brains, a critical organ that requires antioxidant protection from AA [36]. Our xenograft mouse model indicates that VPA administration for 14 days upregulates mSVCT2 expression in the tumor. This upregulation of SVCT2 is crucial, as it likely facilitates increased cellular uptake of AA into cancer cells, as high concentrations of AA are proposed to exert pro-oxidant effects, leading to oxidative damage and cellular stress in tumors [11,48]. Furthermore, co-treatment with AA and VPA for 14 days substantially reduced tumor size and volume (mm^3^) and enhanced the percentage of tumor growth inhibition (%TGI) in the xenograft mouse model. This was accompanied by enhanced expression of the apoptosis-related proteins cleaved caspase-3 and Bax and suppressed expression of pro-caspase 3 and Bcl-2 within the tumor, indicating that the observed antitumor efficacy is mediated through the induction of apoptosis, likely triggered by cellular stress induced by co-treatment with VPA and AA.

Since both VPA and AA are already clinically approved agents [48,49], this therapeutic strategy holds strong potential for clinical translation. Our findings suggest a novel therapeutic strategy of high-dose vitamin C therapy for colorectal cancer, particularly for patients whose tumors exhibit low SVCT2 expression. According to previous research, there is evidence supporting the variability of SVCT2 expression in colorectal cancer patients and colorectal cancer cell lines, with reports showing different expression levels in patient tissue samples and cell lines. This evidence is crucial for vitamin C treatment in considering personalized treatment approaches [50,51]. Therefore, monitoring SVCT2 levels could serve as a valuable biomarker for patient stratification [52], guiding the selection of this combination therapy and optimizing treatment outcomes.

However, our study has some limitations that should be considered. Our in vitro study focused on only two established colorectal cancer cell lines (HCT-116 and DLD-1). However, these studies may not fully represent the heterogeneity observed in patients with cancer. Future studies should validate these findings using a broader panel of colorectal cancer cell lines. Although we observed tumor reduction effects in vivo, the lack of pharmacokinetic data makes it difficult to correlate the administered dose with the actual drug exposure at the tumor site. Therefore, future studies should consider conducting pharmacokinetic analyses for both AA and VPA to establish a more precise relationship between the administered dose and the actual drug exposure. A further major limitation of this study is the lack of patient-derived models, and the reliance on a subcutaneous xenograft model in mice for in vivo studies limits the direct translatability of our findings to the complex tumor microenvironment in real patients. Further research should focus on the anticancer effects of co-treatment with AA and VPA using more physiologically relevant models. Furthermore, identifying the precise target molecules of VPA and elucidating the molecular mechanisms underlying the regulation of SVCT2 expression in cancer cells are required for full understanding. In addition, involving several cancer cell lines that present varying levels of SVCT2 expression to enhance the generalizability of the study results, and including normal epithelial cells as a control group, may provide insight into the specificity of the proposed treatment. These are thereby improving our knowledge of high-dose AA therapies for patients with cancer.

Collectively, our finding indicates that VPA enhanced the synergistic anticancer effects of AA by upregulating SVCT2 expression in low-SVCT2-expressing colorectal cancer cells, suppressing the hormetic dose-response of AA. Notably, to our knowledge, this study is the first to establish that VPA upregulates SVCT2 expression in colorectal cancer cells and to describe the anticancer effects of co-treatment with AA and VPA. Our findings suggest that VPA acts as an SVCT2 inducer, opening the gateway for further research on the potential role of VPA in improving high-dose AA therapy in cancer patients with low SVCT2 expression.

## Data Availability

The original contributions presented in the study are included in the article, further inquiries can be directed to the corresponding authors.

## Abbreviations

The following abbreviations are used in this manuscript:

AA	Ascorbic acid or Vitamin C
VPA	Valproic acid
SVCT2	sodium-dependent vitamin C transporter 2
ROS	Reactive oxygen species
HDAC	Histone deacetylase
C	control
Conc	concentrations
CCK-8	Cell-counting kit-8
Bax	Bcl-2-associated protein x
Bcl-2	B-cell lymphoma 2
GAPDH	Glyceraldehyde 3-phosphate dehydrogenase
shRNA	short hairpin RNA
DCFDA	2′,7′-Dichlorofluorescin diacetate
PI	Propidium Iodide
IP	Intraperitoneal
RTV	Relative tumor volume
%TGI	Tumor growth inhibitor ratio
